# Pattern and Distribution of Skeletal Metastases in Patients With Prostate Cancer in Ghana: A Descriptive Analysis and a Model-Based Digital Nomogram for Oligo-Ostotic Versus Polyostotic Metastases

**DOI:** 10.7759/cureus.91287

**Published:** 2025-08-30

**Authors:** Frank Obeng, Clement Korsah, Mohammed Fadil, Godson Agbeteti, Obed K Amenyo, Eric N Okai, Daniel S Seshie, Jephtha Owusu Boateng, Justice Dzomeku, Nii-Boye Hammond

**Affiliations:** 1 Surgery, University of Health and Allied Sciences, Ho, GHA; 2 Nuclear Medicine, Korle-Bu Teaching Hospital, Accra, GHA; 3 School of Medicine, University of Health and Allied Sciences, Ho, GHA; 4 Perioperative Nursing, Ho Teaching Hospital, Ho, GHA; 5 Information Technology, Ho Polyclinic, Ghana Health Service, Ho, GHA

**Keywords:** axial skeleton, digital nomogram, epidemiology, ghana, nuclear medicine, prostate cancer, skeletal metastasis distribution, staging bone scan, superscan/polyostotic spread

## Abstract

Background: Bone involvement is a frequent and serious consequence of advanced prostate cancer. Understanding the distribution and pattern of bone involvement is essential for early detection, staging, and appropriate therapeutic intervention, particularly in low-resource settings where late presentation is frequent.

Objective: To characterize the anatomical distribution and burden of skeletal metastases in a cohort of newly diagnosed patients with prostate cancer with bone involvement.

Methods: A one-year cross-sectional review of bone scans from 100 newly diagnosed patients with prostate cancer with confirmed skeletal metastases was conducted. Standardized site coding produced frequency tables, total/per-patient counts, and descriptive statistics (mean, median, mode, and IQR). Axial vs. appendicular patterns were shown with bar charts and skeletal heatmaps. A logistic regression model-girded digital nomogram (derived, validated, tested, and updated) predicted superscan/polyostotic metastasis risk. Analyses were in Stata Version 17 (StataCorp LLC, College Station, TX) and Python (Python Software Foundation, Fredericksburg, VA) at α = 0.05.

Results: A total of 470 bone metastatic sites were documented among 100 newly diagnosed patients with prostate cancer, aged 51 to 89 (mean age = 68.81, mode = 65, SD = 7.07 years) with a mean prostate-specific antigen (PSA) of 924.32 ng/ml (range = 5.76 to 2223, SD = 2656.47 ng/ml), alkaline phosphatase (ALP), mean = 239.43, range = 45 to 3265, SD = 438.57, digital rectal examination (DRE) risk (median = 3, IQR = 1), International Society of Urological Pathology (ISUP) risk (median = 3, IQR = 1), and D'Amico risk categories (median = 3, IQR = 0). The number of metastases per patient ranged from 1 to 19, with a mean of 4.7 sites (SD = 2.98), a median of 5 (IQR: 3-7), and a mode of 3. Across the 15 anatomic sites identified, the lesion per-anatomic site per-patient (mean, median, mode, and SD) were 0.31, 1.0, 3.0, and 2.55, respectively. There was notable variability in metastatic burden (solitary lesions in 33.0%, three or more lesions in 51.0%, and superscan/polyostotic lesions (> 4 metastatic sites) in 33.0%), also suggesting heavy burden disease amongst the cohort. The most commonly involved sites were the spine (32.6%), ribs (25.7%), and pelvis (16.1%), the skull (5.2%), and hand and foot bones had zero metastasis. The axial skeleton accounted for 68.1% of all metastatic deposits, and this centripetal spread was further highlighted by heatmap visualizations. Phenotypic clustering revealed that a patient with PSA > 100 ng/ml but moderate ALP (62-122 U/L) is most likely to fall in Q1 (oligo-metastatic pattern; likelihood-ratio (LR) = 31.33 (df = 4), p < 0.0001). Conversely, the combination of age (55-75 years), PSA > 100 ng/ml, and ALP ≥ 222 U/L sharply elevates the likelihood of Q3 (polyostotic disease), regardless of already-high clinical risk scores (LR = 29.77 (df = 4), p < 0.0001). A logistic regression model-girded digital nomogram for oligo-ostotic versus polyostotic metastatic prostate cancer was derived, validated, trained, and deployed, achieving an area under the curve (AUC) of 81.0% at the end.

Conclusion: Axial skeletal metastasis dominates in prostate cancer bone metastasis. Heavy burden metastasis was rampant. These findings emphasize the need for early diagnostic strategies and risk-adapted imaging protocols to improve outcomes in resource-limited settings.

## Introduction

Prostate cancer remains one of the most frequently diagnosed malignancies in men worldwide and is a leading cause of cancer-related morbidity and mortality [1]. In sub-Saharan Africa, including Ghana, late-stage presentation of prostate cancer is common, often resulting in advanced disease with distant metastases at the time of diagnosis [2,3]. Among the common sites of distant spread, the skeletal system is the most frequently involved, with bone metastases occurring in up to 90% of men with advanced or metastatic prostate cancer [4]. These skeletal metastases are not only a marker of disease progression but also a significant source of pain, pathological fractures, spinal cord compression, and decreased quality of life [5]. These make skeletal metastasis a common and morbid sequela of advanced prostate cancer [1-5].

Logothetis and Lin (2005), in their work on the pathophysiology of skeletal metastasis in prostate cancer, reiterate that ‘patients with advanced prostate cancer frequently develop bone metastases' [1]. The tropism of prostate cancer cells for bone and their tendency to induce the osteoblastic phenotype is a result of interactions between prostate cancer cells and osteoblasts [1]. Prostate cancer cells might depend on an osteoblast-derived factor for their growth. Prostate cancer cells produce factors that perturb the bone microenvironment in ways that affect the normal functional balance between osteoblast and osteoclast activities, resulting in osteoblastic metastases [1]. Osteoblasts also secrete factors that facilitate the progression of prostate cancer in bone, and therapeutics designed to target the interaction between prostate cancer and osteoblasts might prevent or treat prostate cancer bone metastases [1]. This sums up their treatise on osteoblasts in prostate cancer metastasis to bone and would undergird substantial portions of the discussions in this paper.

The axial skeleton (skull, spine, and ribs) has been consistently reported as the most affected region due to its rich red marrow content and vascularity, which favor hematogenous tumor cell deposition [6]. The pelvis is a notable site as well. Although this general distribution is widely acknowledged, the specific anatomical pattern, burden, and variation in skeletal metastases among African populations remain poorly described. Such data are critical for refining imaging protocols, staging accuracy, and therapeutic planning, particularly in settings where healthcare resources are limited and the diagnostic pathway is often delayed [7,8].

In Ghana, anecdotal observations from tertiary centers suggest a high prevalence of diffuse skeletal involvement at diagnosis, often manifesting as superscan appearances on bone scintigraphy. However, systematic analyses quantifying and mapping these metastatic patterns are lacking. Moreover, there is a need to assess metastatic burden per patient, identify common skeletal targets, and explore potential clustering of metastases along the axial or appendicular axis. The need for digital nomograms in this respect, derived from data, is also worth mentioning.

This study aims to fill this gap by providing a detailed characterization of the anatomical distribution and site burden of bone metastases among newly diagnosed prostate cancer patients with positive bone scans at a tertiary hospital in Ghana. Through measured statistical analysis, skeletal mapping, and regression analysis leading to the derivation, validation training, and deployment of a digital nomogram for oligo-ostotic versus polyostotic metastasis, we aim to contribute to the global understanding of metastatic spread in prostate cancer and provide contextually relevant data for African oncology practice.

Previous presentation

It is part of a larger Fellowship Degree Thesis (as a partial requirement for the conferment of Fellowship of the Ghana College of Physicians and Surgeons [9]).

## Materials and methods

Study design, setting, and participants

This was a one-year cross-sectional, descriptive analysis, conducted at the Nuclear Medicine and Urology Units of the Korle-Bu Teaching Hospital (KBTH), the largest tertiary referral center in Ghana. The study spanned from April 2018 to March 2019 and involved 100 newly diagnosed, treatment-naïve patients with prostate cancer referred for bone scintigraphy to assess metastatic spread.

Study population

The study included male patients aged 40 years and above with histologically confirmed adenocarcinoma of the prostate who underwent technetium-99m methylene diphosphonate (Tc-99m MDP) bone scans at first diagnosis. Only patients whose bone scans were interpreted as positive for skeletal metastases were included in this analysis.

Inclusion Criteria

Histologically confirmed patients with prostate adenocarcinoma. Treatment-naïve status at the time of bone scintigraphy. Bone scan report indicating the presence of skeletal metastases. Availability of complete medical records and imaging reports.

Exclusion Criteria

Patients with inconclusive or equivocal bone scans. History of previous prostate cancer treatment (e.g., androgen deprivation therapy, chemotherapy, or radiotherapy). Coexisting malignancies with potential for bony spread, known patients with bone disease, fractures, and patients with liver disease, as suggested by elevated liver function tests: aspartate aminotransferase (AST), alanine aminotransferase (ALT), and gamma-glutamyl transferase (GGT).

Data sources, measurements, and variables

Patient data were retrieved from institutional records, including clinical files, pathology reports, and nuclear medicine scan reports. Variables collected included detailed descriptions of bone scan findings, age, prostate-specific antigen (PSA) levels, alkaline phosphatase (ALP) levels, digital rectal examination (DRE) risk grades, International Society of Urological Pathology (ISUP) grading groups, and D'Amico risk classification [9-24]. The primary variable of interest was the anatomical site(s) of bone metastases, as documented in bone scan reports. These free-text reports were manually extracted and standardized. Each anatomical site mentioned (e.g., “L4,” “Right 6th rib,” “skull,” “femur,” etc.) was coded under broader skeletal categories based on anatomical and clinical relevance (e.g., “lumbar spine,” “ribs,” “pelvis,” “femur”). Patients with superscan appearances were categorized as having diffuse skeletal metastases, and all detectable sites listed in the report were coded accordingly.

Quality assurance during the acquisition, reading, and interpretation of ^99^mTc-MDP bone scans for prostate cancer is ensured using end-to-end quality control (QC) measures to preserve diagnostic accuracy [9]. At the outset, radiotracer integrity (≥ 95% labeling), sterility, and proper dosing (740-925 MBq) are confirmed, with hydration and voiding protocols followed. Gamma cameras undergo daily uniformity, weekly energy peak, and periodic resolution and CT attenuation checks. Imaging uses planar scans (10-12 cm/min), motion control (< 1 pixel), and single photon emission computed tomography/computed tomography (SPECT/CT) when needed. Images are checked for Digital Imaging Communication in Medicine-Structured Reporting (DICOM-SR) system completeness, co-registration accuracy, and artifacts. The nuclear physicians analyze planar and SPECT images, applying pattern recognition, lesion-to-normal (L/N) ratios, prior imaging, and clinical markers (PSA, ALP). Structured templates classify lesions as malignant, benign, or equivocal [9]. Equivocal hotspots undergo peer review, targeted SPECT/CT, radiographs, MRI, PET, or follow-up imaging. Biopsy is a last resort. Only after exhausting all tiebreakers is a lesion labeled indeterminate. Regular audits, discrepancy reviews, and DICOM-SR support maintain ≥ 90% sensitivity and ≥ 80% specificity. This structured workflow ensures reliable lesion classification and clinical utility [9].

Bias

In this study, several strategies were employed to minimize bias and enhance the validity of our findings. To begin with, all newly diagnosed prostate cancer patients with confirmed bone metastases and complete clinical and imaging data over the study period were included, reducing the likelihood of selection bias and ensuring that the cohort accurately represented the full clinical spectrum encountered at our institution. Diagnostic consistency was maintained by relying on standardized bone scintigraphy interpretations using uniform nuclear imaging protocols, which helped to minimize misclassification bias.

Independent clinical variables such as PSA, serum ALP, DRE risk classification, ISUP grade, and D’Amico risk categories were categorized based on objective and pre-specified clinical cut-offs, avoiding subjective groupings and enhancing comparability [9-24]. Data collection was performed by trained clinical personnel and independently verified, thereby limiting the potential for information bias. Any discrepancies during data abstraction, if needed, were resolved through consensus to further ensure accuracy.

The burden of bone metastases per patient was stratified using quartile-based thresholds derived from the actual number of distinct skeletal sites involved. This data-driven approach minimized subjective classifications and allowed for a reproducible definition of metastatic load. Minimal missing data were handled transparently; where data were absent, relevant cases were excluded rather than being imputed, thus preventing the introduction of imputation bias.

Blinded statistical analysis was conducted with numerically coded variables and without access to patient identifiers or subjective clinical notes, reducing observer bias. Predictive modeling, such as the logistic regression for superscan status, incorporated an internal validation strategy using a 70:30 data split, which helped guard against overfitting and improved generalizability. Cut-off points for clinical prediction were determined objectively using Youden’s index to balance sensitivity and specificity optimally. Throughout, transparency in coding, variable stratification, and statistical modeling ensured that the analytical process was reproducible and free from analytical bias.

Data collection tool

A structured questionnaire was used for data collection and later transferred into a standardized Microsoft Excel (Microsoft Corporation, Redmond, Washington, United States) spreadsheet developed for the study (Appendix 1). Using the structured questionnaire, we collected data on age, serum total PSA, ALP, DRE, ISUP, and the summarized details of the bone scan findings.

Sample size and sampling

This was a census-based study of all eligible cases over the study period, spanning April 2018 to March 2019. No prior sample size calculation was performed, as the aim was to obtain the full set of cases that meet the eligibility criteria.

Data processing and site classification

The mentioned metastatic sites in the bone scan reports were manually reviewed and grouped into 15 anatomically relevant categories. The axial skeleton included the cervical spine, thoracic spine, lumbar spine, ribs, sternum, and skull, while the appendicular skeleton included the clavicle, shoulder, scapula, humerus, radius/ulna, femur, pelvis, hip, and tibia/fibula. Each patient’s metastasis profile was translated into a list of unique sites, allowing per-patient site counts.

Statistical analysis

Data was analyzed at a 5% significance level, using Stata Version 17 (StataCorp LLC, College Station, TX) for descriptive and regression analysis. Python (Python Software Foundation, Fredericksburg, VA): Pandas, NumPy, SciPy, Matplotlib, and Seaborn libraries were used for skeletal distribution and derivation of the digital nomogram. Descriptive statistics were used to summarize patient characteristics and metastatic site distribution. Frequency distributions were computed for the identified anatomical metastatic sites across the cohort. Per-patient metastasis counts were summarized using measures of central tendency (mean, median), dispersion (range, interquartile range), and frequency (mode, proportions, and percentages). Quartiles and boxplots were generated to illustrate variation in metastatic burden per patient. A bar chart was used to visualize site-specific distribution percentages. A skeletal heatmap was constructed to visually map the burden of metastases across the human skeleton, proportionally scaled to frequency data. A 70:30 partitioning of the data allowed validation, training, and updating of the logistic regression model that was converted to the digital nomogram.

Ethical considerations

Ethical approval for this study was obtained from the Ethical Review Committee of the Korle-Bu Teaching Hospital (Approval number: KBTH-IRB/00086/2018). Patient anonymity was ensured by removing identifiers, and data were handled in accordance with institutional policies and the Declaration of Helsinki.

## Results

Descriptive analysis

A total of 470 bone metastatic sites were documented among 100 newly diagnosed patients with prostate cancer with confirmed bone metastasis on technetium-99 bone scan investigation, aged 51 to 89 (mean age = 68.81, mode = 65, SD = 7.07 years), PSA (mean = 924.32, range = 5.76 to 2223, SD = 2656.47 ng/ml), and ALP (mean = 239.43, range = 45 to 3265, SD = 438.57). DRE risk (median was 3, IQR = 1), ISUP risk (median was 3, IQR = 1), and D'Amico risk categories (median was 3, IQR = 0), Table 1.

**Table 1:** Summary statistics of age, PSA, ALP, ISUP, DRE risk, and D'Amico classification of participants PSA: prostate-specific antigen; ALP: total serum alkaline phosphatase; ISUP: International Society of Urologic Pathology; DRE: digital rectal examination Note: Risk classification is adapted based on published thresholds of PSA, ALP, DRE, ISUP, and D'Amico for bone metastasis prediction [11,18-24].

Variable	Mean	Median	Mode	Min	Max	Range	SD	Q1	Q3	IQR
Age	68.81	69	65	51	89	38	7.07	-	-	-
PSA (ng/ml)	924.32	160	100	5.76	22223	22217.24	2656.47	-	-	-
ALP (IU/L)	239.43	105	100	45	3265	3220	438.57	-	-	-
ISUP	-	3	3	-	-	-	-	2	3	1
DRE Risk	-	3	3	-	-	-	-	2	3	1
D'Amico Risk -		3	3	-	-	-	-	3	3	0

**Figure 1:**
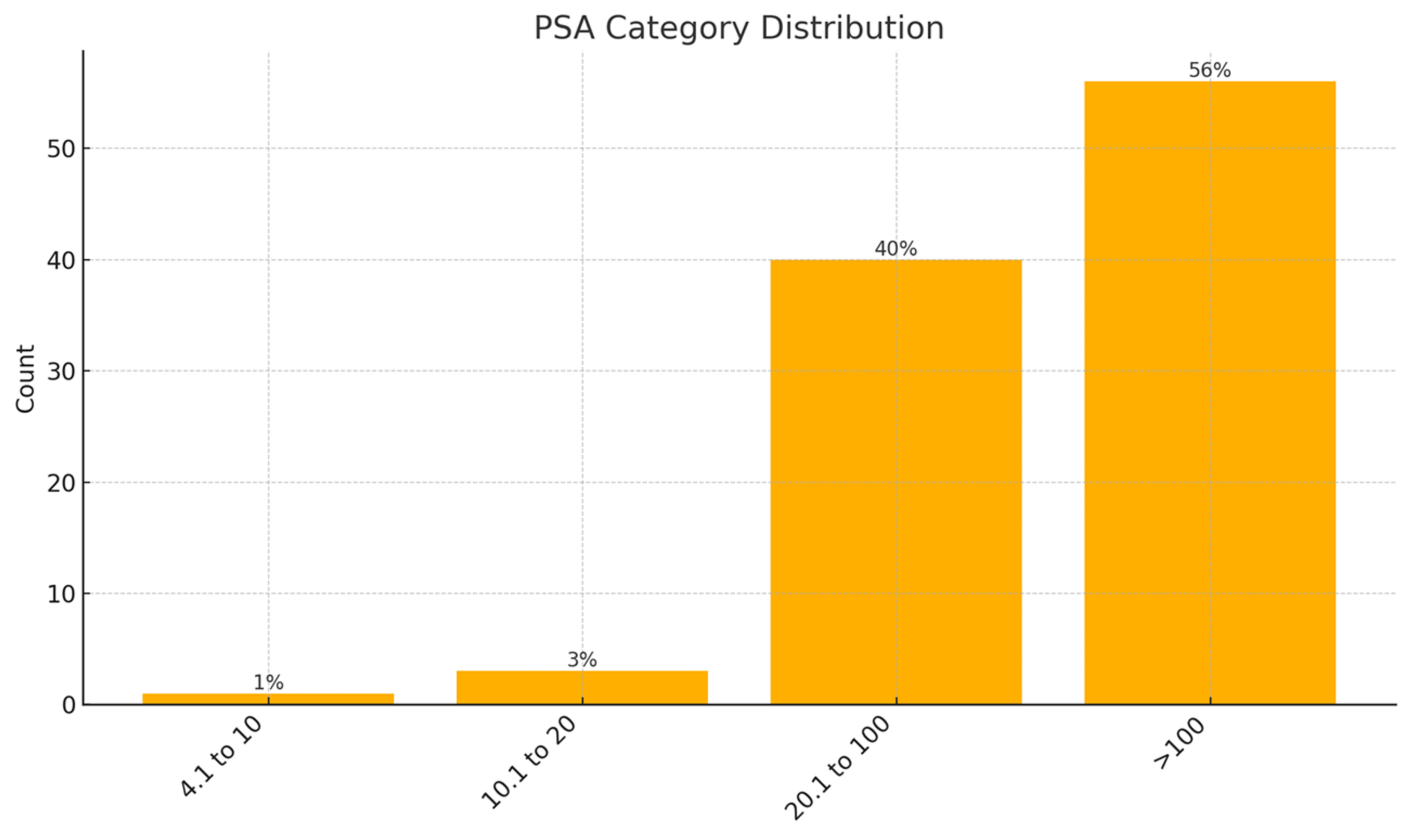
PSA category distribution amongst participants (N = 100) PSA: prostate-specific antigen; units: ng/ml Note: Risk classification is adapted based on published thresholds of PSA for bone metastasis prediction [11,20-24].

**Figure 2:**
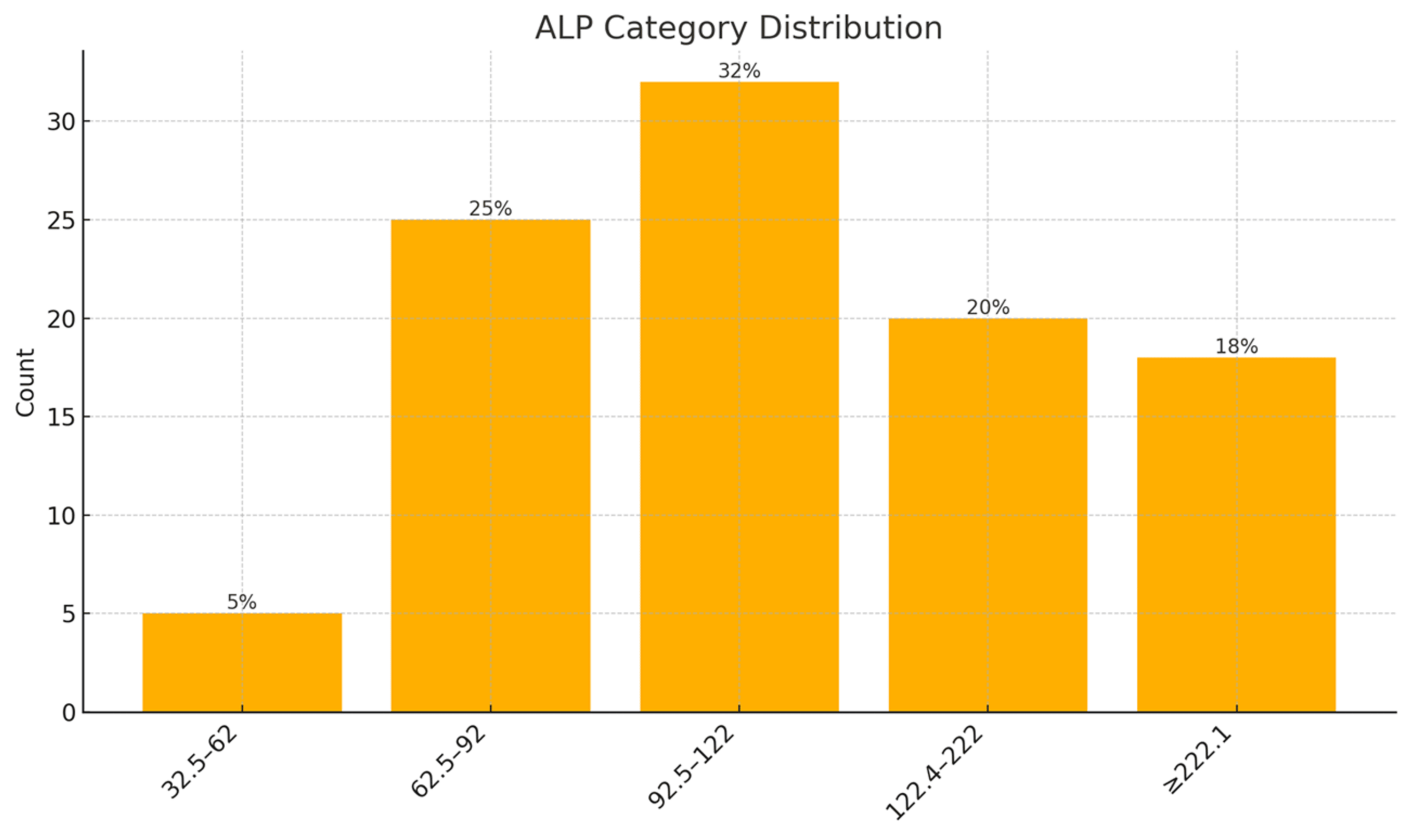
Alkaline phosphatase levels of participants in the study ALP: alkaline phosphatase; units: IU/L Note: Risk classification is adapted based on published thresholds of ALP for bone metastasis prediction [17-19,23,24].

**Figure 3:**
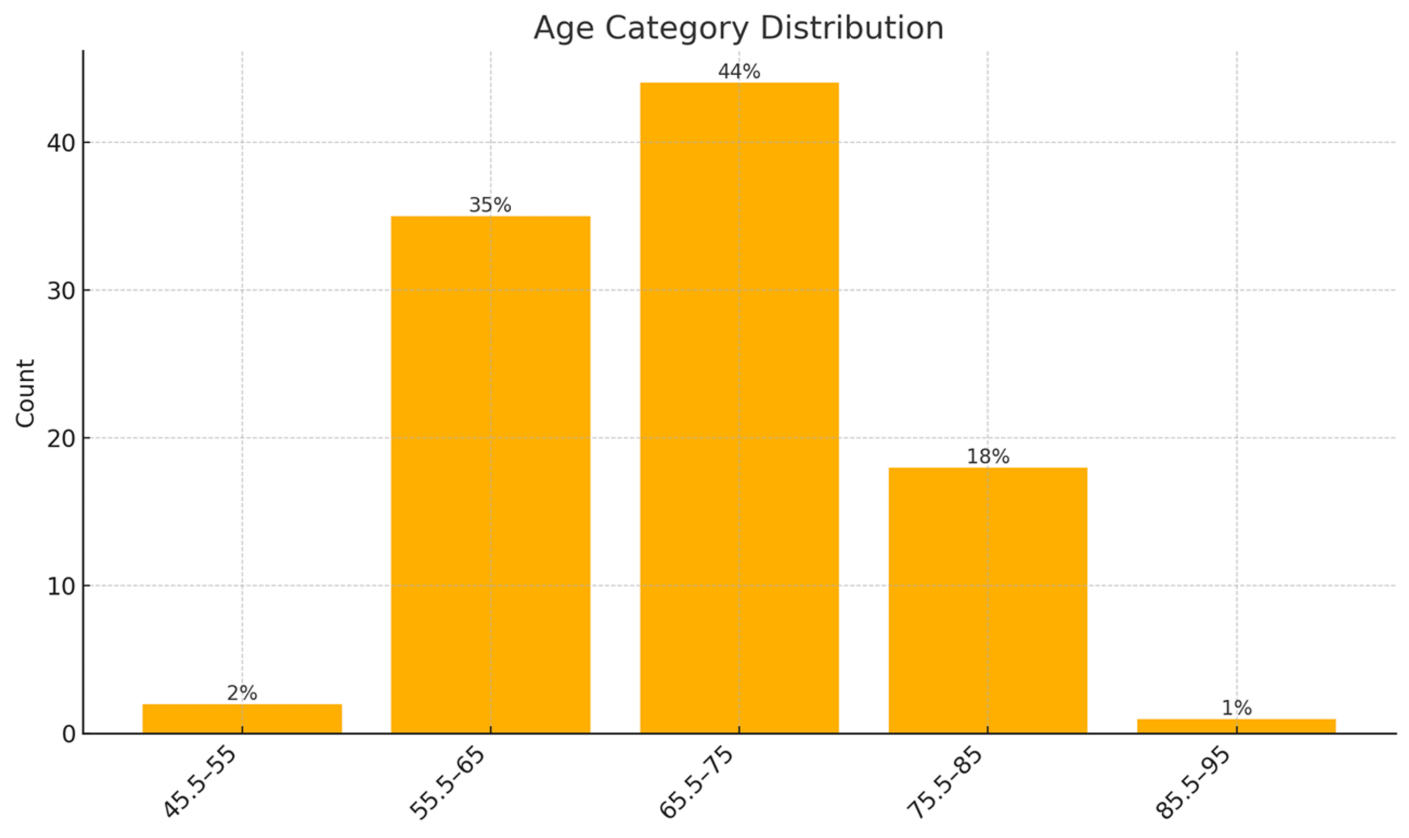
Age categories of participants (in years)

**Figure 4:**
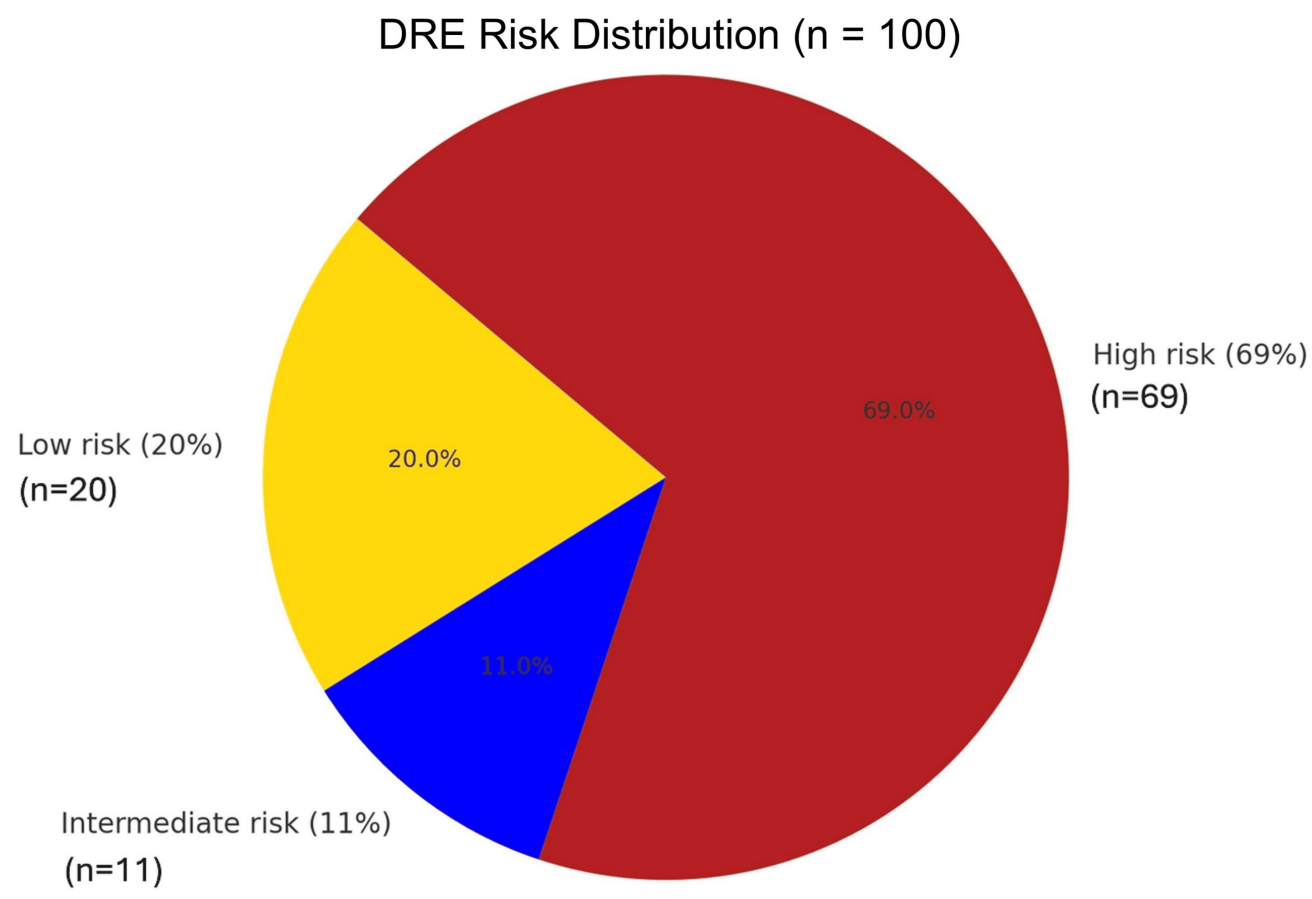
Risk categories of participants, based on digital rectal examination findings, using tumor, node, and metastasis (TNM) disease staging The TNM staging on digital rectal examination can be found in the European Association of Urology (EAU) Guidelines. EANM: European Association of Nuclear Medicine; ESTRO: European Society for Radiotherapy and Oncology; ESUR: European Society of Urogenital Radiology; ISUP: International Society of Urological Pathology (ISUP); SIOG: International Society of Geriatric Oncology; Prostate Cancer Guidelines 2023 [11]. Note: Risk classification is adapted based on the published thresholds of PSA, DRE, ISUP, and D'Amico for bone metastasis prediction [18-24].

**Figure 5:**
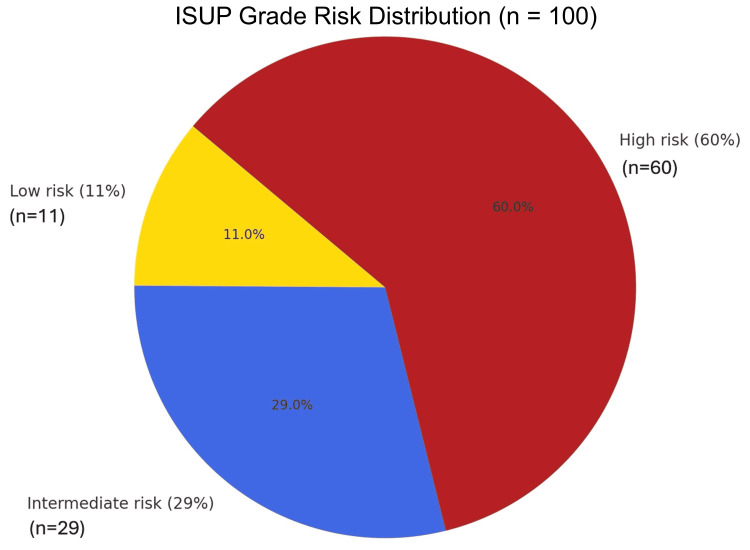
Risk categories of participants based on the International Society of Urologic Pathology (ISUP) grading system, as derived from Gleason scores available from the histopathology results on the prostate core biopsies of the patients in the study cohort (n = 100) Low risk (11%) – ISUP 1 = Gleason 6 (3 + 3), Intermediate risk (29%) – ISUP 2–3 = Gleason 7 (3 + 4 / 4 + 3), High risk (60%) – ISUP 4–5 = Gleason ≥ 8 (4 + 4 / 3 + 5 / 5 + 3) / 9 ( 4 + 5 / 5 + 4) / 10 (5 + 5) The Gleason scoring system and the ISUP scoring system for prostate cancer grading at histology can be found in the European Association of Urology (EAU) Guidelines. EANM: European Association of Nuclear Medicine; ESTRO: European Society for Radiotherapy and Oncology; ESUR: European Society of Urogenital Radiology; ISUP: International Society of Urological Pathology (ISUP); SIOG: International Society of Geriatric Oncology; Prostate Cancer Guidelines 2023 [11]. Note: Risk classification is adapted based on the published thresholds of ISUP and D'Amico for bone metastasis prediction [20-24].

**Figure 6:**
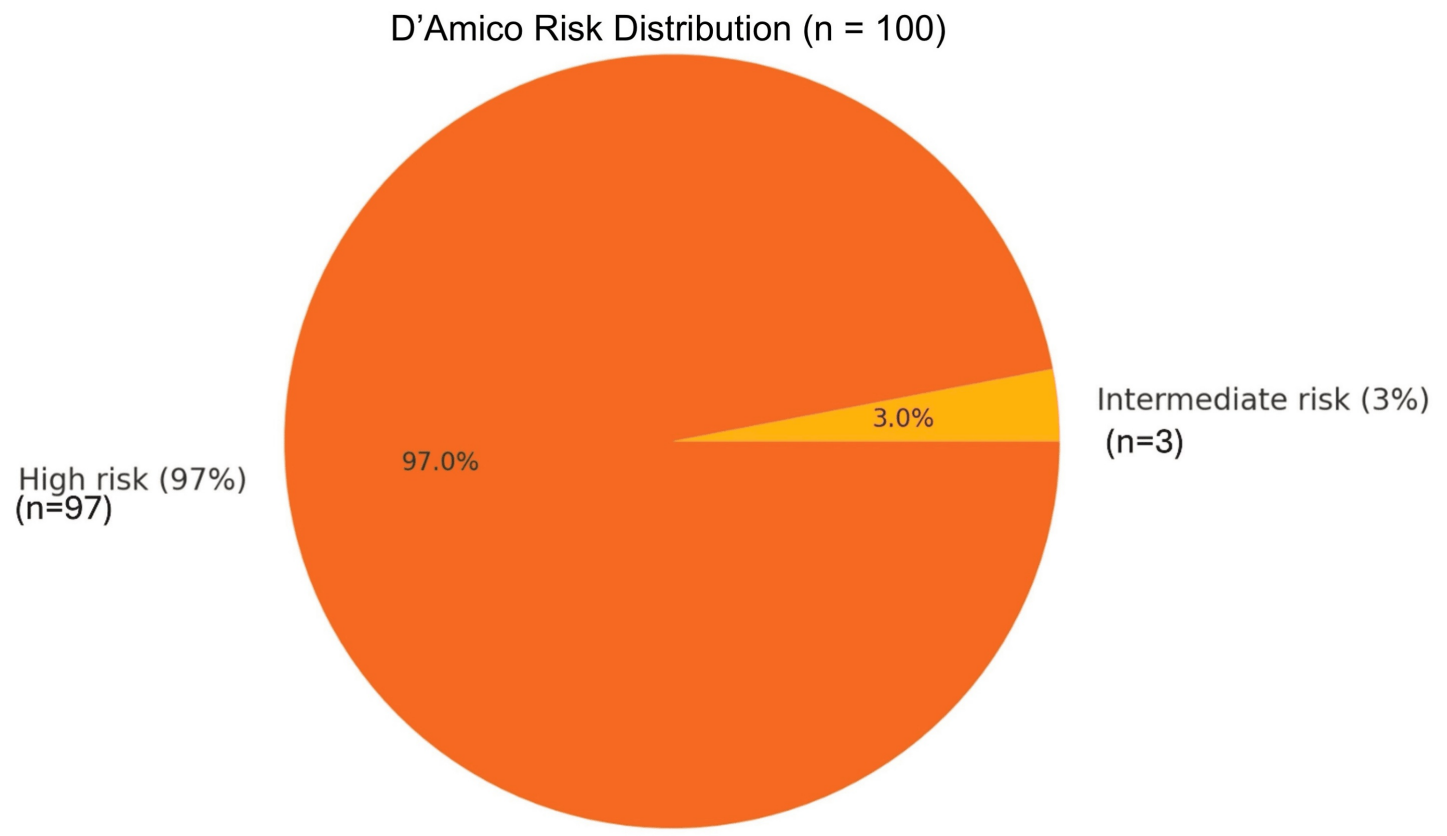
Overall risk stratification of the study participants based on risk aggregation (from PSA, DRE, and ISUP) using the D'Amico recommendations PSA: prostate-specific antigen; DRE: digital rectal examination; ISUP: International Society of Urologic Pathology The D'Amico disease risk stratification for diagnosed prostate cancer can be found in the European Association of Urology (EAU) Guidelines. EANM: European Association of Nuclear Medicine; ESTRO: European Society for Radiotherapy and Oncology; ESUR: European Society of Urogenital Radiology; ISUP: International Society of Urological Pathology; SIOG: International Society of Geriatric Oncology; Prostate Cancer Guidelines, 2023 [11]. Note: Risk classification is adapted based on the published thresholds of PSA, DRE, ISUP, and D'Amico for bone metastasis prediction [20-24].

The most frequent PSA risk group was > 100 ng/ml (56%), confirming that most patients presented with high-burden disease. ALP levels, a marker of skeletal involvement, peaked in the 92.5-122 U/L (32%) and ≥ 222.1 U/L (18%) categories, reinforcing the biochemical signature of bone metastasis. The age group 65.5-75 years was the modal category (44%), aligning with the epidemiologic peak of prostate cancer incidence. Most patients were classified as DRE high risk (69%), ISUP high risk (60.0%), and D'Amico high risk (97%), further highlighting late-stage presentations (Figures 1-6).

Distributional analysis

A*natomical Pattern (Geography and Topology) and Burden (Severity Per Patient) of Skeletal Metastasis in Prostate Cancer*

**Figure 7:**
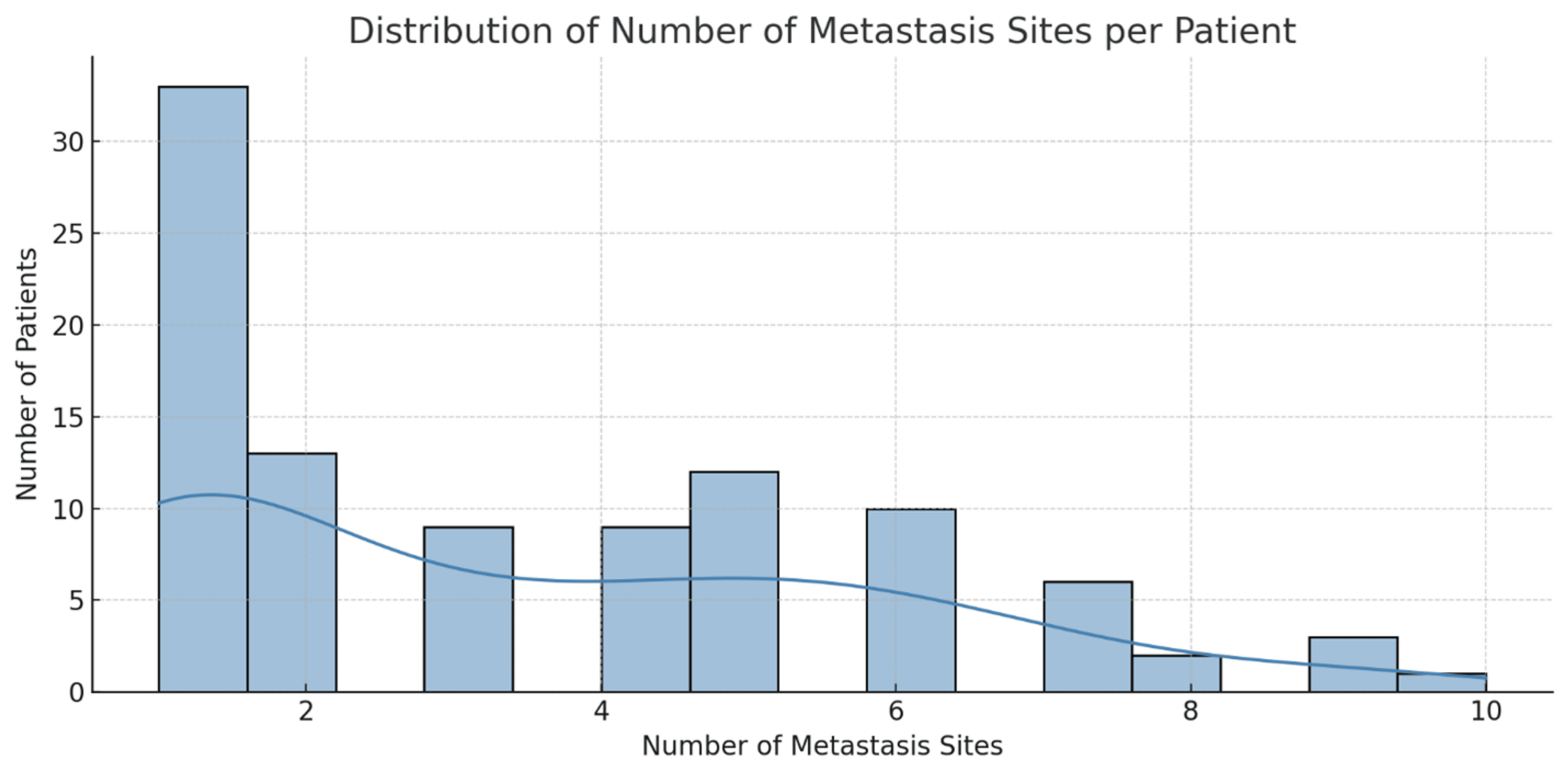
A histogram showing a right-skewed distribution of metastasis counts per patient, emphasizing the presence of patients with extensive skeletal involvement Note: Scoring system for metastasis counts is referred from published scoring systems for bone metastasis burden [14,16,17].

**Figure 8:**
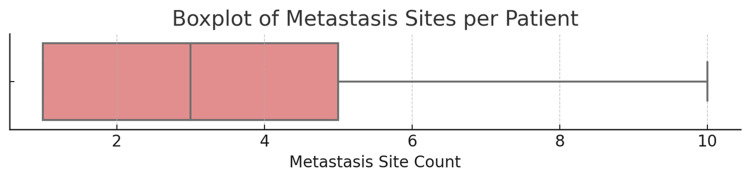
A boxplot showing the distribution of the skeletal metastatic site counts amongst the cohort in the study (N = 100) A boxplot further confirmed central clustering around four to six sites, while also identifying statistical outliers with very high skeletal burden. Note: Scoring system for metastasis counts is referred from published scoring systems for bone metastasis burden [14,16,17].

An extensive review of bone scintigraphy reports from 100 newly diagnosed patients with prostate cancer with bone metastases revealed significant heterogeneity in the distribution and burden of metastatic spread. A total of 470 discrete metastatic sites were identified across all patients, yielding an average of 4.7 bone metastases per individual. The minimum number of metastatic sites per patient was one, while the maximum reached 19, reflecting cases of widespread skeletal involvement. The median number of metastatic sites per patient was four, with an IQR of three to six sites and a modal value of three sites, indicating that oligo-metastatic presentation remains relatively common even within a cohort of advanced-stage patients (Figures 7, 8).

These findings illustrate a diverse but right-skewed distribution of metastatic burden among patients, with a considerable proportion presenting low-volume disease, but a significant proportion exhibiting extensive skeletal involvement. This distribution also reveals a trimodal pattern: one-third of patients presented with solitary lesions, the second one-third had intermediate burden disease (two to four metastatic sites per patient), and the other third had polyostotic disease (> 4 sites), Table 2. The breakdown by quartiles, which revealed that the first quartile (Q1) had three sites and the third quartile (Q3) had six sites, meant that 50% of patients had between three and six metastatic sites. These findings reinforce the urgent need for personalized prognostication and resource-sensitive metastatic workup in similar settings.

**Table 2:** Per patient burden (severity) of skeletal metastasis in prostate cancer amongst the study cohort Note: Scoring system for metastasis counts is referred from published scoring systems for bone metastasis burden [14,16,17].

Metastatic sites	Patients	Percentage
1 site	36	36.0%
2 sites	13	13.0%
3 sites	9	9.0%
4 sites	9	9.0%
>4 sites	33	33.0%

Distribution by skeletal region

**Figure 9:**
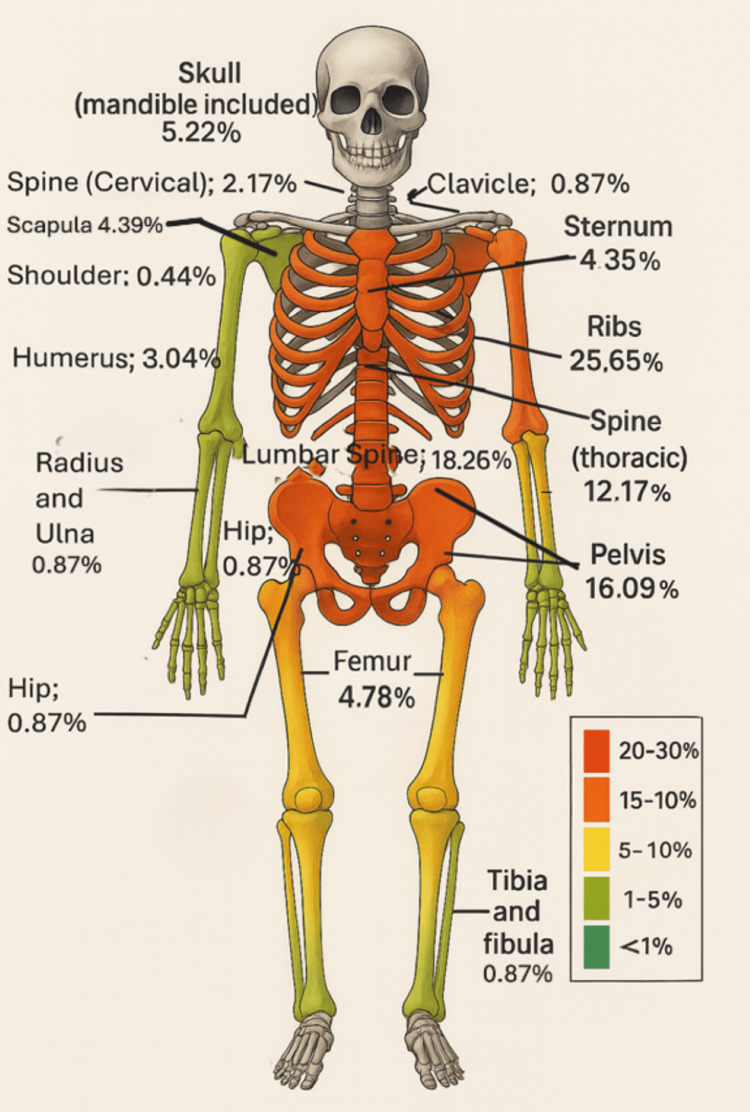
A skeletal heat map of bone metastasis in 100 newly diagnosed treatment-naïve patients with prostate cancer in Ghana, with a total of 470 different lesion sites of skeletal metastasis (N = 470) The skeletal heat map offered a visual appreciation of anatomical hotspots, reinforcing the clinical significance of targeted axial imaging. From the distributional analysis, it can be interpreted that sites with 20-30% (and above) represent very common sites of bone metastasis, sites with 10-20% represent common sites of bone metastasis, sites with 5-10% represent 'not uncommon sites' of bone metastasis, sites with 1-5% represent uncommon sites of bone metastasis, and sites with less than 1% represent rare sites of bone metastasis in this cohort of patients with prostate cancer. Sites like the bones of the hands and feet that showed zero metastasis in this study may be characterized as very rare sites of bone metastasis in prostate cancer. (Source: Authors' original construct, from study data and analysis in the index study). Note: Names or categories of anatomic sites are referred from published work on the skeletal distribution of prostate cancer metastasis [3].

When metastatic sites were classified anatomically, axial skeleton dominance was clearly evident. The ribs were the most commonly affected site, involved in 25.65% of cases. The lumbar spine and pelvis followed closely, involved in 18.26% and 16.09%, respectively. Other frequently involved sites included the thoracic spine (12.17%), skull (5.22%), femur (4.78%), scapula (4.39%), and sternum (4.35%). Less commonly involved sites included the humerus (3.04%), cervical spine (2.17%), and small peripheral bones such as the radius/ulna, tibia/fibula, and clavicle, each contributing under 1% of all recorded metastatic sites. The bones of the hands and feet in this cohort were largely spared of metastasis from prostate cancer (zero spread in those areas), Figure 9.

**Figure 10:**
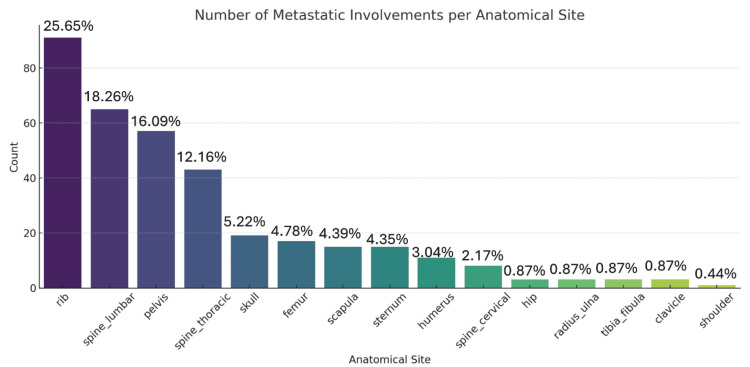
The hierarchy of distribution (counts and percentages) of skeletal metastasis in prostate cancer The total number of metastatic lesion sites over all the 100 study participants is N = 470. Note: The scoring system for metastasis counts is referred from published scoring systems for bone metastasis burden [14,16,17].

**Figure 11:**
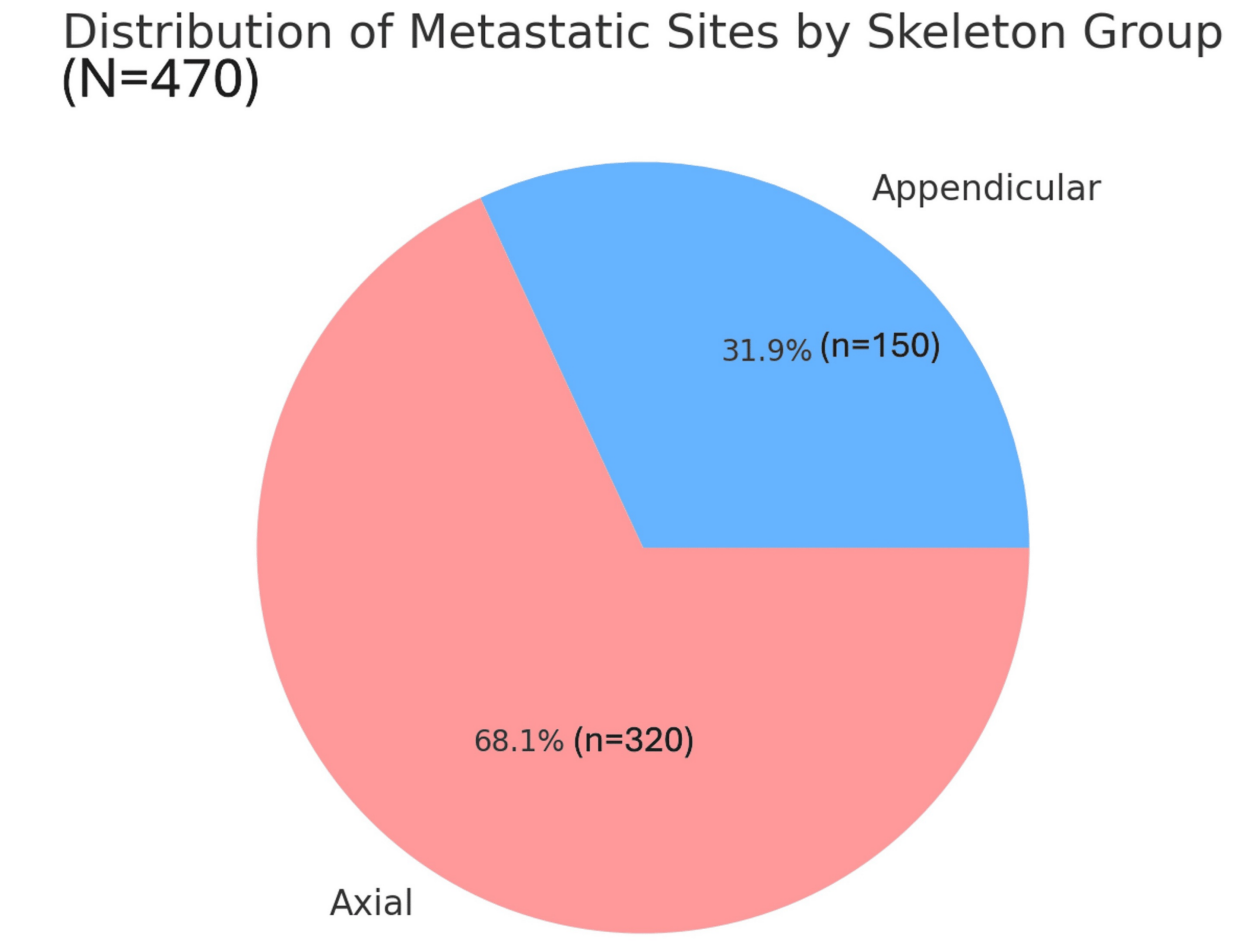
The distribution of skeletal metastasis in the study cohort as aggregated over axial and appendicular skeletal areas Note: Names or categories of anatomic sites are referred from published work on the skeletal distribution of prostate cancer metastasis [3].

The spine together as a unit was involved in 32.60% of all bone metastasis sites in prostate cancer patients in Ghana; the ribs were involved in 25.65%, and the pelvic bones were involved in 16.09%. The three together are involved in the majority (74.34%) of all the bone metastasis sites. In other words, the pelvic bones and lumbar spine together were involved in 34.35% of all the bone metastasis sites, whilst the rib cage (thoracic spine, ribs, and sternum) was involved in 42.17%. The pectoral girdle and upper limb bones (shoulder, clavicle, scapula, humerus, radius, and ulna together) were involved in 9.57% of the sites, whilst head and neck bones together were the sites in a total of 7.39%. The lower limb bones had 5.65% in total, whilst the upper limb bones alone had 3.91%. The bones of the hands and feet were spared. The axial skeleton was involved in 68.1% of the sites, whilst the appendicular skeleton accounted for 31.9% (Figures 9-11).

Axial vs. appendicular classification

A binary classification of metastasis sites revealed that the axial skeleton accounted for over 68.1% of all metastatic deposits, confirming the preferential hematogenous spread of prostate cancer to red marrow-rich areas such as the spine, pelvis, and ribs. The appendicular skeleton, though less commonly involved, featured prominently in cases with superscan appearances or advanced disseminated disease (Figure 11).

Measures of Distribution and Dispersion of Lesions Over Anatomic Sites

Across the 15 skeletal regions assessed, a total of 470 metastatic deposits were recorded. The ribs predominated with 119 lesions, while the mandible and shoulder were affected only once and twice, respectively. The most frequent site-specific count was three lesions, seen in the hip, radius/ulna, tibia/fibula, and clavicle, indicating a strongly skewed distribution. Overall, sites averaged 31.33 lesions (median ≈ 5.2, SD ≈ 27.5), highlighting wide variability in metastatic burden between anatomical locations (Table 3).

**Table 3:** Summary statistics for the individual anatomic sites with metastasis Note: Names or categories of anatomic sites are referred from published work on the skeletal distribution of prostate cancer metastasis [3,16].

Statistic	Value
Total Number of Metastatic Sites	470
Number of Distinct Anatomical Sites	15
Most Frequently Involved Site	Rib (119 mentions)
Least Frequently Involved Site	Shoulder, Mandible (1 and 2 mentioned respectively)
Modal Count (Most Frequent Count)	3 mentions
Sites With Modal Count	Hip, Radius/Ulna, Tibia/Fibula, Clavicle
Mean Count per Site	31.33
Median Count per Site	5.22
Standard Deviation (Site Count)	27.49

Across the 15 anatomic sites identified, the lesions per-anatomic site per-patient (mean, median, mode, and SD) were 0.31, 1.0, 3.0, and 2.55, respectively. 

Clustered bivariate analysis

**Figure 12:**
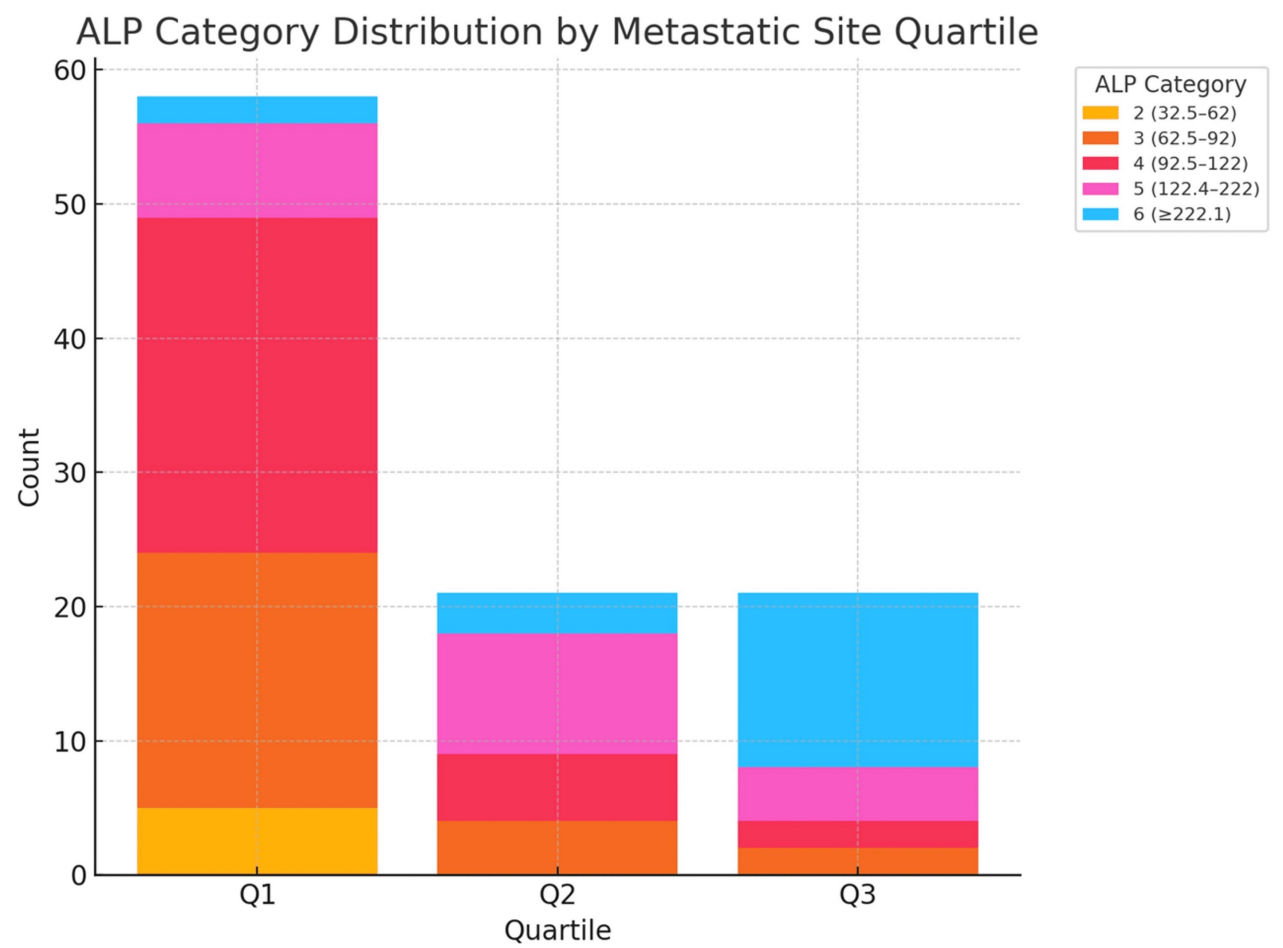
Stacked bar chart showing the various ALP category compositions across Q1–Q3 (clearly demonstrating the shift toward ALP ≥ 222 U/L in Q3) PSA: prostate-specific antigen; ALP: total serum alkaline phosphatase; ISUP: International Society of Urologic Pathology; DRE: digital rectal examination Note: Risk classification is adapted based on published thresholds of PSA, ALP, DRE, ISUP, and D'Amico for bone metastasis prediction [11,18-24]. Metastasis counts are modeled according to clinical imaging patterns [16].

**Figure 13:**
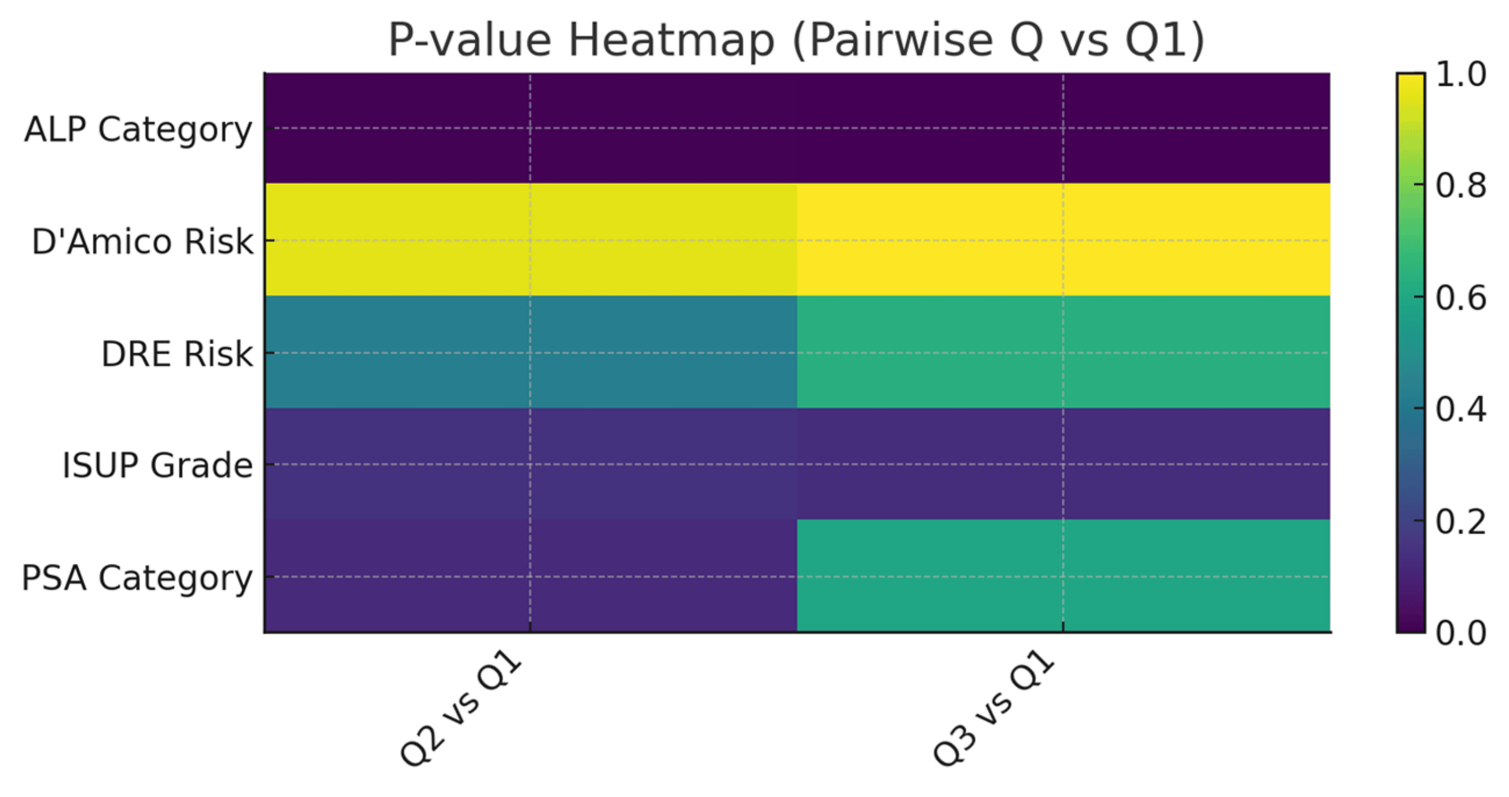
Heat-map showing the pair-wise p-values (Q2/Q3 vs. Q1) for every variable, providing an at-a-glance significance grid PSA: prostate-specific antigen; ALP: total serum alkaline phosphatase; ISUP: International Society of Urologic Pathology; DRE: digital rectal examination Note: Risk classification is adapted based on published thresholds of PSA, ALP, DRE, ISUP, and D'Amico for bone metastasis prediction [11,18-24]. Metastasis counts are modeled according to clinical imaging patterns [16].

ALP elevation is the dominant discriminator of metastatic-burden quartiles; higher categories (≥ 122 U/L and especially ≥ 222 U/L) are markedly over-represented in Q2 and Q3, with highly significant likelihood-ratio (LR) tests (p < 0.0001). PSA, DRE, ISUP, and D’Amico remain heavily skewed toward high-risk strata across all quartiles; consequently, their additional explanatory power is limited, though ISUP shows a weak trend. In practical terms, a patient with PSA > 100 ng/ml but moderate ALP (62-122 U/L) is most likely to fall in Q1 (oligo-metastatic pattern). Conversely, the combination of PSA > 100 ng/ml plus ALP ≥ 222 U/L sharply elevates the likelihood of Q3 (polyostotic disease), regardless of already-high clinical risk scores (Tables 4-6, Figures 1-3, 12, 13).

**Table 4:** Combinations of clinicopathological factors most strongly associated with each quartile of skeletal-metastatic burden (the common phenotypes by metastatic quartile) PSA: prostate-specific antigen; ALP: total serum alkaline phosphatase; ISUP: International Society of Urologic Pathology; DRE: digital rectal examination “Q1–Q3” refer to the three valid quartiles obtained after duplicate bin‐edges were removed (Q1 = lowest number of skeletal sites per patient; Q3 = highest). For every quartile, we list the five most frequent multi-variable phenotypes (PSA × ALP × DRE × ISUP × D’Amico), plus their counts and percentages within that quartile. Together, these phenotypes account for ≈ 70 – 80 % of each subgroup. Note: Risk classification is adapted based on published thresholds of PSA, ALP, DRE, ISUP, and D'Amico for bone metastasis prediction [11,18-24]. Superscan triage bands are modeled according to clinical imaging patterns and net-benefit thresholds [16,25].

Quartile	Phenotype	N	% of quartile
Q1 (1–2 lesions; n = 58)	PSA > 100 ng/ml • ALP 62.5–92 U/L • DRE High • ISUP 3 • D’Amico High	9	15.5 %
-	PSA > 100 • ALP 92.5–122 • DRE High • ISUP 3 • D’Amico High	5	8.6 %
-	PSA 20.1–100 • ALP 92.5–122 • DRE High • ISUP 3 • D’Amico High	4	6.9 %
-	PSA 20.1–100 • ALP 62.5–92 • DRE High • ISUP 3 • D’Amico High	3	5.2 %
-	PSA 20.1–100 • ALP 92.5–122 • DRE Low • ISUP 2 -• D’Amico High	3	5.2 %
Q2 (3–4 lesions; n = 21)	PSA > 100 • ALP 62.5–92 • DRE High • ISUP 3 • D’Amico High	4	19.0 %
-	PSA > 100 • ALP 122.4–222 • DRE High • ISUP 3 • D’Amico High	4	19.0 %
-	PSA > 100 • ALP 92.5–122 • DRE High • ISUP 3 • D’Amico High	3	14.3 %
-	PSA 20.1–100 • ALP 92.5–122 • DRE Int • ISUP 2 • D’Amico High	1	4.8 %
-	PSA 20.1–100 • ALP 122.4–222 • DRE Low • ISUP 3 • D’Amico High	1	4.8 %
Q3 (≥ 5 lesions; n = 21)	PSA > 100 • ALP ≥ 222 U/L • DRE High • ISUP 2 • D’Amico High	4	19.0 %
-	PSA 20.1–100 • ALP ≥ 222 • DRE Low • ISUP 2 • D’Amico High	2	9.5 %
-	PSA 20.1–100 • ALP ≥ 222 • DRE High • ISUP 3 • D’Amico High	2	9.5 %
-	PSA > 100 • ALP 122.4–222 • DRE High • ISUP 3 • D’Amico High	2	9.5 %
-	PSA > 100 • ALP ≥ 222 • DRE High • ISUP 3 • D’Amico High	2	9.5 %

**Table 5:** Statistical strength of association (baseline = Q1) PSA: prostate-specific antigen; ALP: total serum alkaline phosphatase; ISUP: International Society of Urologic Pathology; DRE: digital rectal examination. Likelihood-ratio (LR) chi-square tests compare each higher quartile with the Q1 baseline. Note: Risk classification is adapted based on published thresholds of PSA, ALP, DRE, ISUP, and D'Amico for bone metastasis prediction [11,18-24]. Metastasis counts are modeled according to clinical imaging patterns [16].

Variable (categorical)	Q2 vs. Q1 LR χ² (df) / p	Q3 vs. Q1 LR χ² (df) / p
PSA Category	5.79 (4) / 0.1819	3.49 (4) / 0.6308
ALP Category	31.33 (4) / < 0.0001	29.77 (4) / < 0.0001
DRE Risk	3.55 (3) / 0.4315	1.70 (3) / 0.6326
ISUP Grade	4.29 (2) / 0.2191	3.83 (2) / 0.1462
D’Amico Risk	0.29 (1) / 0.8645	0.00 (1) / 1.0000

**Table 6:** ‘Top 5’ age+clinicopathological combinations per quartile for each skeletal-burden quartile; counts shown interactively PSA: prostate-specific antigen; ALP: total serum alkaline phosphatase; ISUP: International Society of Urologic Pathology; DRE: digital rectal examination Note: Risk classification is adapted based on published thresholds of PSA, ALP, DRE, ISUP, and D'Amico for bone metastasis prediction [11,18-24]. Metastatic counts are modeled according to clinical imaging patterns [16].

Quartile	Age	PSA	ALP	DRE	ISUP	D’Amico	N
Q1 (Lowest Lesion Load)	55.5–65	>100	62–92	High	3	High	5
-	65.5–75	>100	62–92	High	3	High	3
-	65.5–75	20–100	92–122	Low	2	High	3
-	(two others)-		-	-	-	-	-
Q2	65.5–75	>100	92–122	High	3	High	4
-	65.5–75	>100	122–222	High	3	High	3
-	55.5–65	>100	92–122	High	3	High	3
Q3 (Highest Lesion Load)	65.5–75	>100	≥222	High	2	High	4
-	55.5–65	>100	≥222	High	3	High	3
-	65.5–75	20–100	≥222	Low	2	High	2

Most Prevalent Phenotypes of the Age + PSA + ALP + DRE + ISUP + D’Amico Combinations Versus Quartiles of Bone Metastasis Burden

Patterns and clinical takeaways: The age band 55-75 years dominates all quartiles. PSA > 100 ng/ml is ubiquitous, but the discriminating factor is ALP (Q1: ALP mostly 62-122 U/L, Q2: ALP shifts to 92-222 U/L, Q3: ALP ≥ 222 U/L in nearly every leading phenotype).

DRE high and D’Amico high remain common throughout, limiting discriminatory power. ISUP 2-3 separates Q3 (more ISUP 2) from Q1 (mostly ISUP 3), mirroring earlier borderline LR signals.

These combinations refine the phenotype portrait of each metastatic-load tier and can guide triage as follows: patients aged 55-75 with PSA > 100 and ALP ≥ 222 U/L are most likely to harbor polyostotic disease (Q3) and merit immediate whole-body imaging, without allowing them to queue for long periods, Table 6.

Top Anatomical Sites by Metastatic-Burden Quartile

The high-burden quartile (Q3) shows a statistically distinct skeletal pattern-most notably an even greater dominance of rib, spine, and pelvic lesions plus expansion into femur and cervical spine-relative to oligometastatic Q1. These results transition the analysis from per-patient counts to the geography of individual lesions, confirming that metastatic topology/distribution evolves with total lesion load (Table 7).

**Table 7:** Top 10 most frequent anatomic sites per the various quartiles PSA: prostate-specific antigen; ALP: total serum alkaline phosphatase; ISUP: International Society of Urologic Pathology; DRE: digital rectal examination Note: Risk classification is adapted based on published thresholds of PSA, ALP, DRE, ISUP, and D'Amico for bone metastasis prediction [11,18-24]. Metastasis bands are modeled according to clinical imaging patterns [16].

Quartile	Leading skeletal sites (ranked by count)
Q1 (Oligo-Metastatic)	Ribs, Lumbar Spine, Pelvis, Thoracic Spine, Femur, Sacrum, Scapula, Cervical Spine, Humerus, Sternum
Q2	Ribs, Pelvis, Lumbar Spine, Thoracic Spine, Sacrum, Scapula, Femur, Skull, Cervical Spine, Humerus
Q3 (Polyostotic)	Ribs, Lumbar Spine, Pelvis, Thoracic Spine, Sacrum, Femur, Cervical Spine, Humerus, Scapula, Skull

The LR (chi-square) tests for the global test (all sites × quartiles) reveal interesting patterns: A global LR χ² test (χ² = 263.84, df = 168, p < 0.001) confirms that the skeletal distribution of metastases varies significantly across lesion-count quartiles. Specifically, Q3 differs from Q1, based on the pairwise Q3 vs. Q1 site distribution (χ² = 195.59, df = 129, p = 0.0001), corroborating substantial shifts in anatomical involvement between these strata (Table 7).

Multivariate Analysis

After stepwise forward multivariate regression analysis, the initial regression model equation (with PSA, ALP, ISUP, DRE, D'Amico, and Model A) is presented below (Equation 1):

\(\operatorname{logit}(P_B) = \begin{array}{l} -3.928 + 2.273 \cdot \mathbf{1}(\text{ALP} \geq 222) + 1.589 \cdot \mathbf{1}(122 \leq \text{ALP} < 222) \\ + 0.813 \cdot \mathbf{1}(92.5 \leq \text{ALP} < 122) + 0.564 \cdot \mathbf{1}(62.5 \leq \text{ALP} < 92.5) \\ + 0.092 \cdot \mathbf{1}(32.5 \leq \text{ALP} < 62.5) + 0.991 \cdot \mathbf{1}(\text{PSA} > 100) \\ + 0.684 \cdot \mathbf{1}(20.1 < \text{PSA} \leq 100) + 0.377 \cdot \mathbf{1}(10.1 < \text{PSA} \leq 20) \\ + 0.183 \cdot \mathbf{1}(4.1 < \text{PSA} \leq 10) + 0.982 \cdot \mathbf{1}(\text{DRE high}) \\ + 0.571 \cdot \mathbf{1}(\text{ISUP} = 3) + 0.613 \cdot \mathbf{1}(\text{D'Amico high}) \end{array}\)

**Figure 14:**
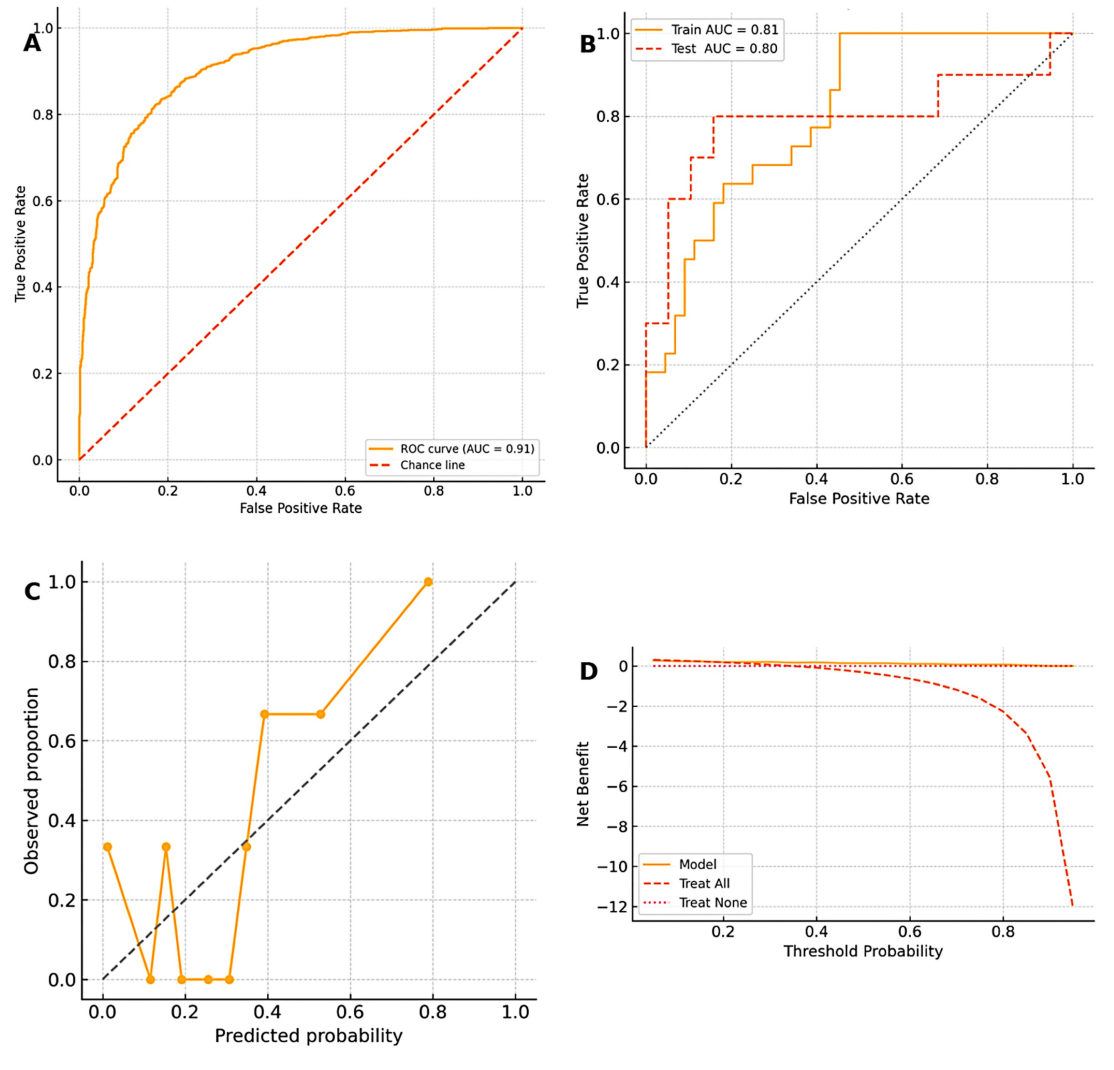
Receiver-operator-characteristic (ROC) curves for models pre- and post-validation; calibration plots and decision curve analysis post-validation A: ROC for the model at the development phase; B: ROC curves for train (training and updating of the model) and independent test (external validation); C: calibration plots - the post-validation, post-training model calibration plot (Model B), the model is well-calibrated, and for the Hosmer-Lemeshow,  p = 0.63; D: decision-curve analysis (DCA) demonstrating the clinical utility of the predictive model for identifying superscan metastasis/polyostotic bone disease in newly diagnosed patients with prostate cancer (independent test set). The graph illustrates the net clinical benefit of using the predictive model across a range of threshold probabilities for deciding whether to perform further intervention based on the likelihood of a superscan result. The y-axis represents the net benefit, which balances the number of true positives against the harm of false positives at each decision threshold. The x-axis represents the probability threshold at which clinicians would choose to intervene. Three clinical strategies are compared: Model (solid amber line): selectively intervening in patients whose predicted probability of superscan metastasis exceeds the threshold. Treat All (dashed orange line): assuming all newly diagnosed patients with prostate cancer have superscan disease and intervening universally. Treat None (dotted pink line): assuming none have superscan disease and withholding intervention entirely. The model supports risk-adapted stratification of patients for appropriate diagnostic and therapeutic planning. Note: Risk classification and variable categorization are adapted based on published thresholds of PSA, ALP, DRE, ISUP, and D'Amico for bone metastasis prediction [11,18-24]. Superscan triage bands are modeled according to clinical imaging patterns and net-benefit thresholds [16,25].

Tables 8, 9, and Figure 14 summarize model odds ratios, leading to coefficients, as well as their performance and evaluation (internal validation) metrics.

**Table 8:** The odds-ratio highlights for the model equation PSA: prostate-specific antigen; ALP: total serum alkaline phosphatase; ISUP: International Society of Urologic Pathology; DRE: digital rectal examination Alkaline phosphatase (ALP) ≥ 222 U/L increases the odds of a superscan ~10-fold, independent of other high-risk features, and is therefore the key driver. Note: Risk classification is adapted based on published thresholds of PSA, ALP, DRE, ISUP, and D'Amico for bone metastasis prediction [11,18-24]. Metastasis counts are modeled according to clinical imaging patterns [16].

Predictor *(reference category in italics)*	Odds ratio (OR)	95% confidence interval (CI)	P-value	Statistical test used	Test statistic (z)
ALP ≥ 222 U/L vs. *ALP 32–62*	9.7	1.7 – 55.8	0.01	Logistic Regression	2.56
ALP 122–222 vs. *ALP 32–62*	4.9	0.8 – 29.9	0.094	Logistic Regression	1.68
PSA > 100 vs.* PSA 4–20*	1.4	0.3 – 6.2	0.64	Logistic Regression	0.47
ISUP High Risk vs.* ISUP Low Risk*	2.5	0.5 – 11.7	0.24	Logistic Regression	1.17
DRE-High Risk vs. *DRE-Low Risk*	1.1	0.3 – 4.2	0.91	Logistic Regression	0.11
D’Amico-High Risk vs.* Intermediate Risk*	1.3	0.2 – 6.9	0.78	Logistic Regression	0.28

**Table 9:** Summary metrics and confusion matrix values for the SuperScan (> 4 bone sites) prediction model PSA: prostate-specific antigen; ALP: total serum alkaline phosphatase; ISUP: International Society of Urologic Pathology; DRE: digital rectal examination; ROC: receiver operating characteristic; AUC: area under the curve Note: Risk classification is adapted based on published thresholds of PSA, ALP, DRE, ISUP, and D'Amico for bone metastasis prediction [11,18-24]. Metastasis counts are modeled according to clinical imaging patterns [16].

Metric	Statistical test/formula	Model development stage	Model validation and updating stage
Youden's Index	J = Sensitivity + Specificity - 1	0.50	0.37
Accuracy	Accuracy = (TP+TN)/(TP+FP+TN+FN)	0.84	0.87
Precision	TP / (TP + FP)	0.75	0.80
Recall (Sensitivity)	TP / (TP + FN)	0.64	0.67
F1 Score	2 × (Precision × Recall) / (Precision + Recall)	0.69	0.73
ROC AUC	Area under the ROC curve	0.91	0.80
Confusion Matrix - Actual No / Predicted No	Count-	30	-
Confusion Matrix - Actual No / Predicted Yes	Count-	4	-
Confusion Matrix - Actual Yes / Predicted No	Count-	8	-
Confusion Matrix - Actual Yes / Predicted Yes	Count-	14	-

The model demonstrates superior net benefit across a clinically relevant threshold range of approximately 10% to 70%, indicating that its use is preferable to treating all or treating none in decision-making regarding bone scan-based staging or further management. It shows excellent discrimination; even with categorical inputs, it captures the strong biochemical signal of ALP in predicting heavy skeletal tumor burden. We then proceeded to partition the data into 70:30 and conducted external validation with 30% of the dataset after developing the model with 70% of the dataset. The whole model was then trained and updated with the complete dataset available. The outcomes are summarized in Table 9 and Figure 14.

The final continuous model after train-test validation (Model B), with intercept and betas coming from the L2-regularized logistic fit (ridge regression) on the 30% training set, is presented in Equation (2) as follows:

\(\begin{equation} \operatorname{logit}(P_{B}) = -3.2183 + 0.1826 \ln(\text{PSA} + 1) + 0.7349 \ln(\text{ALP} + 1) + 0.2501 \,\text{DRE}_{\text{num}} + 0.2184 \,\text{ISUP}_{\text{num}} + 0.0958 \,\text{D'Amico}_{\text{num}} \tag{2} \end{equation}\)

During the external validation and training processes, we trained a predictive model using 30% of the patients. To make sure the model doesn't "memorize" the data and works well on new cases, we added a mathematical control (L2 regularization) to keep it balanced and reliable.

The Youden’s optimal cut-off, derived from the 70% training dataset, came to PYouden=0.37 (the Youden index itself was 0.50). So, we use P ≥ 0.37 as the operating point for maximal combined sensitivity and specificity. The Hosmer-Lemeshow chi-square statistic was 6.12 (df = 8), with a p-value of 0.634, showing a good fit with the data.

The clinical triage interpretation based on model-derived thresholds suggests a graduated approach to imaging and treatment decisions [25], Table 10. For patients with predicted probabilities below 0.20, bone scans should still be conducted to exclude metastasis, but intensive or high-cost imaging such as prostate-specific membrane antigen (PSMA) PET may be unnecessary due to the low likelihood of superscan disease; at this threshold range, the model performs similarly to a “treat-none” approach.

**Table 10:** Interpretation of model decision cut-off points for clinical triage PSA: prostate-specific antigen; ALP: total serum alkaline phosphatase; ISUP: International Society of Urologic Pathology; DRE: digital rectal examination; PSMA: prostate-specific membrane antigen Note: Risk classification and variable categorization are adapted based on published thresholds of PSA, ALP, DRE, ISUP, and D'Amico for bone metastasis prediction [11,18-24]. Superscan triage bands are modeled according to clinical imaging patterns and net-benefit thresholds [16,25].

Threshold band	Suggested action	Net-benefit context
< 0.20	Still conduct the bone scanning, but avoid intensive bone imaging; low likelihood of superscan	Model ≈ “treat-none” line
0.20 – 0.37	Consider ALP repeat or targeted imaging	Small net-benefit gain
≥ 0.37 (Youden)	Recommend full-body bone scan ± PSMA PET; high superscan risk	Highest combined sensitivity & specificity
≥ 0.50	Presume heavy disease; prioritise systemic therapy	Net benefit plateaus

Between 0.20 and 0.37, the likelihood of superscan involvement modestly increases, warranting consideration of a repeat ALP test or more targeted imaging to clarify disease extent. In this intermediate range, the net clinical benefit begins to rise, albeit gradually.

At probabilities ≥ 0.37-the Youden index threshold, the model achieves optimal sensitivity and specificity. This justifies recommending a full-body bone scan, possibly complemented by PSMA PET, to evaluate the patient for widespread skeletal metastases, given the high superscan risk.

Beyond a 0.50 probability, the disease burden is presumed to be heavy, and systemic therapy should be prioritized. While imaging remains important, the model's net benefit plateaus here, indicating diminishing returns from further diagnostic escalation (Table 10 and Figure 14). The decision curve analysis [25], which suggests that the model is beneficial, is depicted in Figure 14D.

The model has been converted to a digital risk calculator (nomogram) using Python (a statistical tool) as the tool for packaging and deployment, and is available online. It could help clinicians gauge the severity or extent of bone spread of cancer in patients with prostate cancer who have already been determined to be high risk for metastasis by the Korle-Bu ALP, PSA, and bone metastasis risk calculator.

The links (two links) to the ‘Superscan Status Present’ versus ‘No Superscan’ digital risk calculator can be found in references [26] and [27].

There is also an MS Excel version (Microsoft Corporation, Redmond, Washington, United States) of that same calculator. Its contents and how it works are as follows: it is an Excel sheet with the final model formula programmed into it. To use it, enter raw PSA value (ng/ml) and ALP (U/L), clinical scores for DRE, ISUP, and D'Amico risk stratification. Hidden columns convert to log values; cell F2 returns the superscan probability [28].

## Discussion

Summary of findings

In this cohort of 100 newly diagnosed patients with prostate cancer, bone metastases were both frequent and widely distributed, with a total of 470 metastatic deposits identified. The metastatic burden was substantial, with one-third of patients presenting with solitary lesions, over half with three or more lesions, and another third exhibiting extensive polyostotic (“superscan”) disease. The axial skeleton (particularly the spine and ribs) and pelvis were the predominant sites of involvement, accounting for more than two-thirds of all lesions, while no metastases were detected in the small bones of the hands or feet. Patterns of spread followed a centripetal distribution, most pronounced in the lumbar and thoracic spine. Phenotypic clustering demonstrated that lower ALP levels alongside markedly elevated PSA were linked to oligo-metastatic disease, whereas higher ALP (ALP ≥ 222 U/L) in the setting of PSA >100 ng/ml was strongly associated with polyostotic involvement, independent of conventional clinical risk scores. These patterns informed the development of a logistic regression-based digital nomogram to discriminate between oligo- and polyostotic disease, which achieved strong predictive discrimination (AUC = 81.0%) upon validation.

Comparison with existing literature

Bone metastasis remains a defining feature of advanced prostate cancer, profoundly influencing prognosis, therapeutic choices, and quality of life. In this cohort of 100 newly diagnosed patients with prostate cancer, a total of 470 skeletal metastases were documented, highlighting a significant metastatic burden at presentation [1-3]. The patients, predominantly aged between 51 and 89 years (mean 68.8 years), reflect the typical demographic profile for advanced disease [4,5]. Notably, the mean PSA level was markedly elevated (924.3 ng/ml), with an exceptionally wide range and large standard deviation, indicative of heterogeneous tumor biology and varied disease progression at diagnosis. Similarly, the mean ALP level was substantially raised (239.4 U/L), consistent with high skeletal turnover and metastatic bone activity [1-5]. Median values for DRE, ISUP, and D’Amico categories were all at the highest levels (median = 3), with narrow interquartile ranges, suggesting that the majority of patients presented with clinically advanced and high-risk disease [20-24]. These findings underscore the predominance of aggressive prostate cancer phenotypes in this population, likely due to delayed diagnosis and limited access to early screening, and reinforce the need for intensified early detection strategies and tailored management approaches in high-burden settings [4,5].

In our cohort of patients with skeletal involvement, metastatic spread followed the classic “centripetal” pattern, favoring red-marrow-rich regions of the axial skeleton, most commonly the ribs (25.7%), lumbar spine (18.3%), pelvis (16.1%), and thoracic spine (12.2%). This distribution mirrors the well-described osteotropism of prostate adenocarcinoma, driven by reciprocal signaling between tumor cell integrins, chemokines, and the bone marrow microenvironment and the favorable chemokine-adhesion molecule gradients [1,2].

The dominance of axial skeletal involvement (68.1%) observed in our series is consistent with the seminal work by Bubendorf et al. (done in the year 2000), who reported bone metastases in 90% of autopsy-proven advanced prostate cancer cases, predominantly involving the spine, ribs, and pelvis [3]. This large autopsy series by Bubendorf et al. involved 1,589 cases. Furthermore, the rib involvement rate among our cohort exceeds many Western reports, yet is biologically plausible and clinically relevant: careful clinico-radiological correlation excluded post-traumatic or degenerative uptake, supporting true metastatic disease. While some studies downplay rib metastases as often traumatic or non-specific [10], the meticulous clinical correlation during our study supports the pathologic relevance in most cases.

Regional or racial variation is also evident: Hirobe et al. observed predominant lumbar and pelvic lesions with comparatively less rib involvement in a retrospective study in Japanese patients [4]. This may suggest differences in presentation [11,12], probably related to pathophysiologic variability in marrow distribution or differences in imaging technique/interpretation protocols [4,11].

The skeletal metastatic burden at the individual level was considerable. Patients harbored a mean of 4.7 (median 5.2; range 1-19) metastatic sites. Comparable African series report a mean of 3.2 lesions [5], whereas Western cohorts, benefiting from earlier PSA-based screening, often present with lower counts [6]. The modal value of three sites and the finding that only 30% of men had < 3 lesions highlight a sizeable subgroup with potential “oligometastatic” disease. In the era of PSMA PET and metastasis-directed therapy, accurate lesion counting is increasingly pivotal for stratifying low- versus high-volume disease and tailoring curative-intent approaches [1,13,14]. Nevertheless, our maximum of 19 lesions per patient, frequent superscan appearances (33.0%), and the slight skew of the data in favor of heavy burden disease (42.0% with four or more lesions, or 51.0% with three or more lesions), underscore the parallel reality of extensive, late-presenting disease in resource-limited settings [15]. Superscan patterns with diffuse skeletal tracer uptake and absent renal visualization are typically linked to heavy tumor burden and poor outcomes [7,16]. This emerging reality of a heavy burden of polyostotic metastasis amongst our prostate cancer patients may make us rethink the need for PSA-based early screening as a proactive national policy to avoid such large numbers of heavy burden diseases in our population (and match that with aggressive but properly tailored therapy [1]). This, in the long run, may help us bridge our yawning disparities. The required policy leap may come with its own need for substantial resource inputs, and the risk of overdiagnosis can also not be discounted, but the benefit in the long run may justify the change.

Appendicular involvement was uncommon, femur 4.8%, scapula 4.3%, humerus 3.0%-and almost exclusively accompanied widespread axial disease or superscan findings, corroborating observations that appendicular deposits signal late-stage, treatment-resistant progression [8].

Beyond anatomic mapping, our data reinforces important health-system implications. Late presentation and high polyostotic burden likely reflect limited awareness, PSA testing, and specialist access across sub-Saharan Africa [15]. Rational, risk-adapted use of bone scintigraphy remains essential: indiscriminate scanning in low-risk men wastes scarce resources and risks false positives, whereas underutilization delays accurate staging [11]. Integrating conventional bone scans with emerging PSMA-based modalities may offer cost-effective algorithms for African centers [11]. The findings of this study affirm the aggressive nature and late-stage detection of prostate cancer in this population, with high frequencies of biochemical and radiological markers of advanced disease [9-24]. The combined statistical and visual exploration delivers strong foundational insight for quality improvement in the areas of clinical audits, documentation, training of all corps of medical practitioners, and policy planning. It also underscores the need for comprehensive skeletal assessment in newly diagnosed patients with prostate cancer in sub-Saharan Africa, where late presentation remains common. The findings also reinforce the importance of risk-adapted bone scan strategies, as overuse in low-risk cases may lead to false positives, while underuse in resource-limited settings can delay definitive care.

It is important to add that the consistently high affinity of prostate cancer for bone mediated by osteoblast activation and a “vicious cycle” of reciprocal tumor-bone signaling [1,2] suggests that biological therapies targeting this interface (e.g., radioligand therapy, bone-modifying agents) may hold particular promise for our patient population.

The predictive model for polyostotic metastasis and its digital deployment

A digitally deployed nomogram grounded in a multivariable logistic regression model [26-28] now enables rapid distinction between oligo- and polyostotic skeletal disease in newly diagnosed prostate cancer [18-25]. With an internally and externally validated AUC of 91.0% and 81.0%, respectively, the tool offers clinically useful discrimination: patients whose predicted probability exceeds the calibrated threshold can be triaged immediately to whole-body imaging or systemic therapy (including chemo-hormonal and bone-seeking radionuclides), whereas those below the cut-point may undergo bone scans on a reasonably rapid appointment basis [17,25]. Embedding the nomogram in the nuclear-medicine workflow could also provide an objective cross-check for bone-scan interpretations: the nuclear physician can compare the model-predicted burden against visual impression, flagging discordant cases for secondary review and thereby standardizing report quality. Beyond day-to-day triage, aggregated nomogram outputs can feed departmental dashboards, allowing quality-control audits of reporting accuracy and facilitating continuous calibration as imaging technology and patient profiles evolve [17].

Limitations of the study

This study is not without limitations. First, its single-center design may limit the generalizability of the findings to broader populations, particularly in settings with differing diagnostic resources or patient demographics. It was a one-year cross-sectional study, and this may limit the comprehensiveness of the data. Additionally, the reliance on bone scintigraphy, while widely available, may underestimate or misclassify metastatic sites compared to more advanced imaging modalities like PSMA PET/CT. The lack of follow-up or survival data precludes assessment of prognostic outcomes, and our categorization of superscan status based solely on-site count, though practical, may not fully capture the functional or clinical impact of diffuse skeletal involvement. Furthermore, while biochemical markers like PSA and ALP were incorporated, other potential prognostic variables such as lactate dehydrogenase (LDH) or visceral metastases were not evaluated, which may limit the comprehensiveness of risk stratification. We also want to stress the absence of prognostic follow-up in this cohort. 

## Conclusions

This study highlights the extensive skeletal metastatic burden in newly diagnosed patients with prostate cancer within our cohort, with a predominant tropism for the axial skeleton, especially the ribs, lumbar spine, pelvis, and thoracic spine. In practical terms, a patient with PSA > 100 ng/ml but moderate ALP (62-122 U/L) is most likely to fall in Q1 (oligo-metastatic pattern). Conversely, the combination of PSA > 100 ng/ml plus ALP ≥ 222 U/L sharply elevates the likelihood of Q3 (polyostotic disease), regardless of already-high clinical risk scores. By delineating the distribution of skeletal metastases, this study provides critical insights for optimizing palliative interventions, improving risk stratification, and prioritizing resource allocation in low-resource environments. 

We have identified a pattern of late presentation and widespread skeletal involvement, reflective of potential diagnostic delays and limited early screening in our setting. The high frequency of polyostotic disease and superscan appearances further emphasizes the need for prompt diagnostic strategies, improved staging protocols, and context-specific imaging guidelines. This reality of heavy burden disease, evidenced by at least 33% with superscans/polyostotic disease, should make us rethink the need for PSA-based early screening as a proactive national policy to avoid such large numbers of heavy burden diseases in our population (and match that with aggressive but properly tailored therapy). This, in the long run, may help us bridge our yawning disparities. There is a heterogeneous but quantifiable (bimodal/trimodal) pattern of skeletal dissemination in newly diagnosed Ghanaian men with prostate cancer. These findings underscore the dual clinical realities of a sizeable oligometastatic subgroup amenable to emerging focal therapies and a substantial fraction with extensive disease requiring aggressive systemic or palliative care. Integrating precise lesion quantification with clinical and biochemical risk markers and deploying context-appropriate imaging algorithms (including the new Superscan Digital Risk Calculator derived from this study) will be critical for personalized management and optimal resource allocation in low-resource settings, such as ours. The need for larger multicenter studies (going forward) to broaden the evidence base to support these recommendations cannot, however, be discounted.

## Appendices

Appendix 1

**Table 11:** Study questionnaire and data-abstraction form PSA: prostate-specific antigen; ALP: total serum alkaline phosphatase; DRE: digital rectal examination; SPECT/CT: single photon emission computed tomography/computed tomography; PSMA: prostate-specific membrane antigen This questionnaire was sourced and modified from the study questionnaire in our previous publication in the Cureus Journal [17]. It is freely available, as it is open source.

Section	Code	Variable / field
SECTION A: Administration	A1	Study ID
SECTION A: Administration	A2	Date (DD-MM-YYYY)
SECTION A: Administration	A3	Data abstractor initials
SECTION B: Demographics & Background	B1	Age at diagnosis (yrs)
SECTION B: Demographics & Background	B2	Ethnicity (1=Akan 2=Ewe 3=Ga-Dangme 4=Northern 5=Other)
SECTION B: Demographics & Background	B3	Region of residence
SECTION B: Demographics & Background	B4	Family history of PCa (0=No 1=Yes 9=Unk)
SECTION C: Baseline Clinical & Laboratory	C1	Serum PSA (ng/mL)
SECTION C: Baseline Clinical & Laboratory	C2	Serum ALP (U/L)
SECTION C: Baseline Clinical & Laboratory	C3	Bone pain (0=No 1=Yes)
SECTION C: Baseline Clinical & Laboratory	C4	DRE T-stage (T1 - T4)
SECTION C: Baseline Clinical & Laboratory	C5	DRE-risk stratum (1 - 3)
SECTION C: Baseline Clinical & Laboratory	C6	Systemic symptoms (tick): Wt loss, Anaemia, Fracture, None
SECTION C: Baseline Clinical & Laboratory	C7	Gleason pattern: Primary + Secondary
SECTION C: Baseline Clinical & Laboratory	C8	ISUP-numeric grade (1 - 5)
SECTION C: Baseline Clinical & Laboratory	C9	D’Amico-risk stratum (1=Low 2=Int 3=High)
SECTION D: Imaging	D1	Modality (1=Planar 2=SPECT/CT 3=PSMA-PET 4=Other)
SECTION D: Imaging	D2	Date of scan (DD-MM-YYYY)
SECTION D: Imaging	D3	Superscan reported (0=No 1=Yes)
Anatomical Site Grid (Presence 0/1, Count)	E1	Skull (incl mandible)
Anatomical Site Grid (Presence 0/1, Count)	E2	Cervical spine
Anatomical Site Grid (Presence 0/1, Count)	E3	Thoracic spine
Anatomical Site Grid (Presence 0/1, Count)	E4	Lumbar spine
Anatomical Site Grid (Presence 0/1, Count)	E5	Sacrum/Pelvis
Anatomical Site Grid (Presence 0/1, Count)	E6	Ribs
Anatomical Site Grid (Presence 0/1, Count)	E7	Sternum
Anatomical Site Grid (Presence 0/1, Count)	E8	Humerus
Anatomical Site Grid (Presence 0/1, Count)	E9	Radius + Ulna
Anatomical Site Grid (Presence 0/1, Count)	E10	Femur
Anatomical Site Grid (Presence 0/1, Count)	E11	Tibia + Fibula
Anatomical Site Grid (Presence 0/1, Count)	E12	Scapula / Shoulder
Anatomical Site Grid (Presence 0/1, Count)	E13	Clavicle
Anatomical Site Grid (Presence 0/1, Count)	E14	Hip (GT)
Anatomical Site Grid (Presence 0/1, Count)	E15	Total lesions
Anatomical Site Grid (Presence 0/1, Count)	E16	Axial count
Anatomical Site Grid (Presence 0/1, Count)	E17	Appendicular count
